# Delayed fatal neurotoxicity in post CAR-T cell therapy for multiple myeloma, a case report

**DOI:** 10.1016/j.lrr.2026.100577

**Published:** 2026-03-03

**Authors:** Hamza Khoudari, Abdalla Shoaib, Muhammad Nashatizadeh, Nausheen Ahmed, Forat Lutfi, Muhammad Mushtaq, Leyla Shune, Anurag Singh, Sunil Abhyankar, Joseph McGuirk, Haitham Abdelhakim

**Affiliations:** a University of Debrecen, Debrecen, Hungary; b Division of hematologic Malignancies and Cellular therapeutics, University of Kansas Medical Center, Kansas City, KS 66160, Wyandotte; c Department of neurology, University of Kansas Medical Center, Kansas City, KS 66160, Wyandotte

**Keywords:** Multiple myeloma, CAR-T, ICANS, B-cell malignancy, Immunotherapy

## Abstract

Anti-BCMA CAR T-cell therapy, specifically ciltacabtagene autoleucel, has significantly improved outcomes for relapsed/refractory multiple myeloma (RRMM). While early-onset immune-effector cell–associated neurotoxicity syndrome (ICANS) is a recognized complication, delayed-onset and non-ICANS neurological syndromes, such as movement and neurocognitive toxicity (MNT), present unique diagnostic and therapeutic challenges.

We report a case of a 61-year-old female with a 15-year history of RRMM who developed severe, delayed neurotoxicity 50 days after ciltacabtagene autoleucel infusion. The clinical course began with confusion and rapidly progressed to grade 4 ICANS characterized by lethargy, rigidity, and parkinsonian features. Serial MRI imaging revealed evolving, symmetric T2/FLAIR hyperintensities in the basal ganglia and brainstem, and reactive pachymeningitis. Despite aggressive multi modal immunosuppression, the patient’s condition remained refractory and expired on day 93. Post-mortem autopsy confirmed severe bilateral hippocampal sclerosis, diffuse gliosis, and microglial infiltration.

This case highlights a fatal presentation of delayed neurotoxicity that overlaps with the emerging MNT phenotype. As CAR-T therapies expand, this case underscores the necessity for prolonged clinical vigilance and the urgent need for novel management strategies for refractory, late-onset neurotoxicity.

## 1 Introduction

Multiple myeloma is a common hematological malignancy that remains largely incurable despite therapeutic advances [1]. B cell maturation antigen (BCMA) is heavily expressed on mature B lymphocytes, plasma cells and plasma blasts, but not on CD34+ hematopoietic cells making it an ideal therapeutic target with a lower risk of on target off tumor toxicity [2]. Anti-BCMA CAR-T cell therapy is considered a highly efficacious option in relapsed or refractory multiple myeloma (RRMM) and is associated with improved overall survival, yet early toxicities such as cytokine-release syndrome (CRS) and immune-effector cell–associated neurotoxicity syndrome (ICANS) are frequent [3,4]. While ICANS traditionally emerges within 1–2 weeks post-infusion, delayed cases over 4 weeks) are increasingly recognized. A global registry study reported 52 delayed events, some occurring more than 100 days post-treatment [5]. While classical ICANS, presents earlier within 1–2 weeks, improves with corticosteroid based immunosuppression and is dominated by cortical symptoms. Non-ICANS neurological syndromes, particularly movement and neurocognitive toxicity (MNT), typically follows a delayed, progressive course, are often refractory to treatment and are characterized by basal ganglia involvement [6].

Non-ICANS ranges from mild cognitive, executive dysfunction, parkinsonian features, postural instability, language deficits to severe encephalopathy, cerebral edema and very rarely, brain herniation [[6], [7], [8], [9], [10]]. The pathophysiology of neurotoxicity is complex and multifactorial. Inflammatory cytokines (notably IL-6, TNF-α, and VEGF) increase endothelial permeability, destabilize the BBB, facilitate the infiltration of further cytokines and immune cells into the CNS, triggering neuroinflammation and neuronal dysfunction [11,12]. There is a debate regarding the degree of BCMA expression in the human brain. BCMA is reported to be expressed in basal ganglia mainly in the caudate and putamen and plays a role in neural development [[13], [14], [15]]. A recent study showed that its expression is low and suggests a low risk of on-target off-tumor cytotoxicity [16]. However, Parkinson-like symptoms following anti-BCMA CAR T-cells have been reported which further support the clinically significant expression of BCMA receptors in the basal ganglia [14,17].

ICANS management options remain limited beyond high dose steroids especially in refractory cases [18]. Grading scales, such as ICE grading scales, aid in stratifying the severity of ICANS and guiding therapeutic interventions [19]. Nonetheless, challenges persist in distinguishing ICANS from other neurological, toxic or infectious complications, especially when it occurs late in the CAR-T cell therapy course highlighting the importance of comprehensive clinical evaluation and multidisciplinary collaboration.

### 1.1 Case presentation

**Fig. 1:**
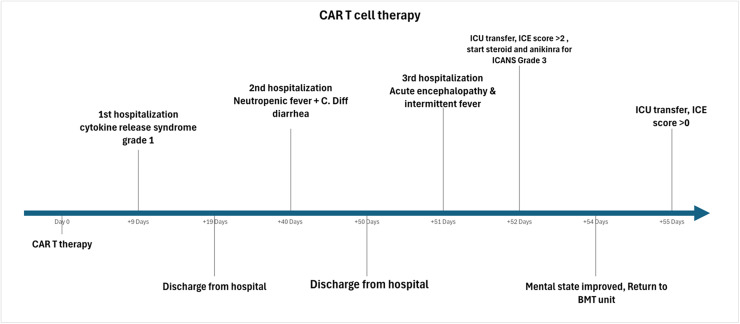
Timeline of major case events.

A 61-year-old female with a 15-year history of relapsed progressive lambda light chain multiple myeloma (MM) who underwent anti-BCMA CAR-T cell therapy with ciltacabtagene autoleucel. Her prior treatments included multiple lines of therapy VAD (vincristine, doxorubicin, dexamethasone), TD (thalidomide, dexamethasone), VDT (bortezomib, dexamethasone, thalidomide), and RVD (lenalidomide, bortezomib, dexamethasone) in addition to two autologous hematopoietic stem cell transplants. She progressed biochemically on a regimen of daratumumab, pomalidomide, and dexamethasone (DPd) prior to CAR T-cell therapy. The bone marrow biopsy showed 43% monoclonal plasma cells while no PET avid extramedullary lesions on PET scan. The M protein was 0.04 gm/dl and lamda chain was 200.52 mg/dl with kappa/lamda <0.01 prior to CAR-T cell therapy. She received ciltacabtagene autoleucel CAR-T after lymphodepletion with fludarabine and cyclophosphamide. The CAR-T cell course is summarized in Fig. 1.

Her CAR-T therapy course was complicated with cytokine release syndrome grade 1 (CRS) on day +9 in the form of fever, and hypotension with maximum grade 2 within 8 hours. She required ICU transfer and received vasopressors briefly with rapid improvement of hypotension after administration of tocilizumab and corticosteroids. However, she had persistent fever for 5 days and infectious work up including cerebrospinal fluid (CSF) on day +15 analysis was unremarkable (Table 1). The absolute lymphocyte counts (ALC) peaked at 2.53 k/ul on day +16 post CAR-T therapy with subsequent decline. Her hospitalization lasted 10 days and she was discharged home with close monitoring in the clinic. She achieved complete remission on work up at day +30 but had persistent pancytopenia.

**Table 1:** Summary of cerebrospinal fluid (CSF) studies per day performed post CAR-T cell infusion.

CSF analysis	Day +15	Day +51	Day +56	Day +58	Day +63
Protein mg/dl	30	82	114	114	171
WBCs cells/μL	0	37	40	24	8
RBCs cells/μL	95	29	12	6	158
Lymphocytes %	100	90	100	97	72
Neutrophils %	0	2	0	0	0
Monocytes %	0	8	3	3	28
Clarity	clear	clear	clear	clear	clear

**Fig. 2:**
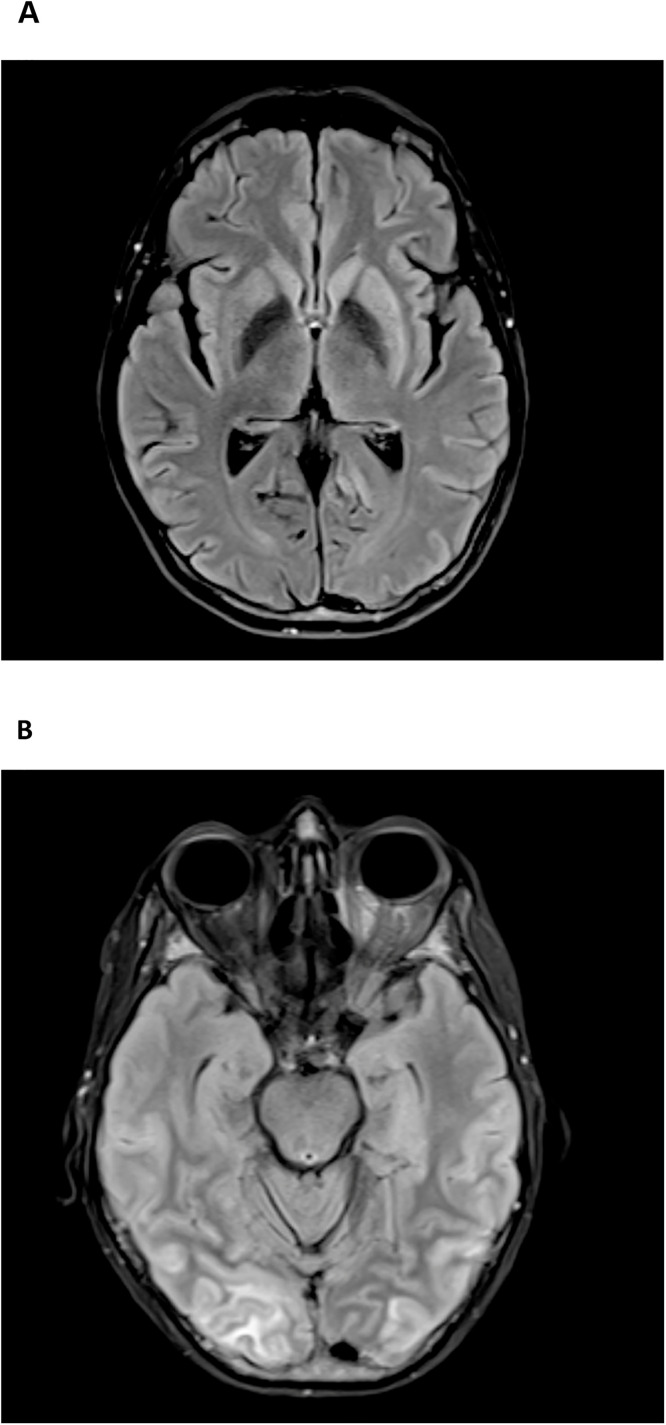
MRI findings. 2A: representative MRI slice showing T2 FLAIR hyperintense signal abnormality within the left greater than right caudate and putamen basal ganglia without evidence of superimposed enhancement. 2B: Representative MRI slice showing T2 FLAIR early signs of PRES syndrome.

On day +40, she was admitted with neutropenic fever and diarrhea and was found to have C. difficile infection. The fever and diarrhea subsided, and she was discharged on day +50, but next day (day +51), she was readmitted with recurrent fever and newly developed confusion, her ALC showed mild increase and peaked at 2.6 k/ul on day +51. Given the concern for delayed ICANS, high-dose steroids were initiated alongside broad-spectrum antimicrobials, and a repeat infectious evaluation was initiated. CSF analysis showed elevated protein and WBCs (predominantly lymphocytes) but was negative for infections (Table 1). Initial MRI brain showed changes resembling parkinsonism with abnormal T2 hyperintensity in the left greater than right caudate and putamen without abnormal enhancement (Fig. 2A).

On day +52, her mental status continued to deteriorate with an ICE score of 2 prompting initiation of anakinra, escalation of the high-dose corticosteroid and transfer to the ICU for close monitoring. She had initial clinical improvement and transferred out of the ICU on day +54 with ICE score 7. Subsequently, on day +55 she became increasingly lethargic and rigid on examination, progressed to ICANS grade 4 (ICE 0), necessitating intubation. Repeat MRI showed unchanged basal ganglia findings. EEG showed moderate diffuse background slowing with superimposed left temporal focal slowing with no changes following levetiracetam loading. Lumbar puncture again showed elevated protein and lymphocytic pleocytosis (Table 1), with negative viral PCR, bacterial and fungal cultures, and autoimmune antibody panel. Repeat MRI on day +58 revealed cerebral cortical and juxtacortical flair hyperintensity which are consistent with early posterior reversible encephalopathy syndrome (PRES) alongside cerebral edema and subdural fluid collections and T2 flair hyperintense signal abnormality within the caudate and putamen symmetrically on both sides without superimposed enhancement (Fig. 2B). EEG was negative for seizures but showed severe encephalopathy with continuous 2.5-3 Hz periodic triphasic waves during periods of arousal and generalized theta/delta activity. In the following days different immunosuppressive modalities were administered including intravenous immunoglobulin (IVIG) (days +57-60), intrathecal chemotherapy (days +58, +63), and continued high-dose corticosteroid and anakinra.

She received anti-thymocyte globulin (ATG) for 3 days starting day +69 given persistent neurological condition with no improvement. Serial imaging showed some improvement in the cortical edema but worsening of basal ganglia and brainstem involvement. On day +73, MRI revealed development of subdural hygromas and diffuse pachymeningeal thickening, concerning for reactive pachymeningitis. Repeated CSF analysis showed elevated protein and lymphocytic predominance without evidence of infection (Table 1). Continued monitoring of her lab values showed persistent elevation of ferritin since early post-infusion, peaking around day +10 (supplementary Fig. 1)

Last MRI obtained on Day +86 showed improvement of pachymeningeal thickening and subdural effusion but persistent symmetric basal ganglia flair hyperintensity involving putamen, caudate and now the brain stem. Given the lack of improvement, elevated neuron specific enolase, on day +93 her family made the decision to transition to comfort measures. The patient expired rapidly following palliative extubation. A postmortem autopsy showed severe bilateral hippocampal sclerosis with diffuse gliosis and infiltration of microglial/macrophages. Similar evidence of neuronal loss and gliosis were found in the other parts of neocortex with variable degrees.

## 2 Discussion

This case illustrates the presentation of delayed neurotoxicity on Day +50 after anti-BCMA CAR-T infusion in a patient with relapsed/refractory multiple myeloma. The onset was delayed but acute, refractory. Delayed onset ICANS s reported as late as 100 days post infusion, suggesting that it may be underrecognized rather than truly rare [20,21]. The patient manifested no neurological symptoms until Day +50. Due to the delayed presentation, alternative causes such as infections, chemotherapy side effects, and autoimmune etiologies were considered. Extensive infectious workups, including blood, urine, CSF, and imaging of the chest and abdomen, were repeatedly negative during her prolonged admission. Fludarabine-induced neurotoxicity was considered as a potential contributor to her progression, however, absence of hallmark features of fludarabine toxicity, such as visual deficits, ataxia, or demyelinating changes on imaging or on autopsy, made this diagnosis unlikely [22]. Furthermore, inflammatory syndromes such as Hemophagocytic lymphohistiocytosis and macrophage activation syndrome were ruled out with serum ferritin levels of <1000 and no other positive criteria.

Even though the patient developed neurotoxicity several weeks after CRS improved, the prior CRS episode may still have contributed. CRS is associated with systemic inflammation and endothelial dysfunction, which can weaken the blood–brain barrier. These changes could explain the evolving MRI from parkinsonism-like basal ganglia abnormalities, followed by PRES and later reactive pachymeningitis. This radiographic evolution paralleled the patient’s fluctuating neurologic symptoms, reduced seizure threshold, and episodes of autonomic instability.

MRI findings were also notable for progressive, symmetric T2/FLAIR hyperintensity in the basal ganglia and brainstem without enhancement. Although non-specific, these findings are consistent with immune-mediated neuroinflammation. Notably, the basal ganglia involvement aligns with recent data suggesting expression of BCMA in these regions, raising the possibility of on-target off-tumor neurotoxicity as a contributing mechanism [14]. Moreover, the patient's Parkinson-like features, including rigidity, further support this mechanism.

Importantly, the patient’s presentation also overlaps with recently recognized non-ICANS neurological complications following BCMA targeted CAR-T therapy. In particular, movement and neurocognitive toxicity (MNT), that is characterized by delayed neurocognitive decline and hypokinetic movement disorder or parkinsonism, often accompanied by basal ganglia involvement on neuroimaging [22]. The combination of delayed onset, progressive encephalopathy, rigidity, and symmetric caudate/putamen T2/FLAIR hyperintensity in our patient is suggestive of this emerging phenotype. Therefore, while the syndrome fulfilled criteria for severe immune-effector cell–associated neurotoxicity and was managed as delayed ICANS, MNT represents a key overlapping entity for BCMA-CAR-T neurotoxicity that should be considered. Notably, this patient had prior biopsy-proven extramedullary disease (pancreatic and adrenal involvement), which has been proposed as a potential risk factor for non-ICANS neurotoxicity after BCMA-targeted CAR T-cell therapy, but the patient had no PET avid lesions on PET scan at the time of CAR-T therapy. However, supporting data remains limited.

While the low disease burden does not exclude severe CAR-T toxicity, the concomitant *C. difficile* infection may have worsened the inflammation and triggered further CAR-T cell activation against normal cells expressing BCMA at a low level. Another factor is receiving metronidazole which disrupts the gut microbiota and can potentially modulate the inflammatory environment [[23], [24], [25]].

This report highlights a delayed and fatal case of ICANS, presenting 50 days post-CAR-T cell therapy initiation in a patient with refractory multiple myeloma. The clinical course, imaging, and post-mortem findings support a diagnosis of immune-mediated neurotoxicity, possibly involving on-target off-tumor effects. This case emphasizes the necessity for vigilant monitoring and a high index of suspicion for ICANS, even beyond the typical window of onset. As the use of CAR-T cell and bispecific T cell engagers therapies expands, there is critical need for development of diagnostic and prognostic biomarkers for ICANS in addition to novel therapeutic strategies to improve outcomes for patients.

## Informed consent

Written informed consent was obtained from the patient’s as part of treatment consent at the University of Kansas Medical Center for the publication of a de-identified information and any accompanying images.

## CRediT authorship contribution statement

**Hamza Khoudari:** Writing – review & editing, Writing – original draft, Visualization, Validation, Resources, Methodology, Formal analysis, Data curation, Conceptualization. **Abdalla Shoaib:** Writing – review & editing, Writing – original draft, Visualization, Validation, Resources, Methodology, Formal analysis, Data curation, Conceptualization. **Muhammad Nashatizadeh:** Writing – review & editing, Visualization, Validation, Investigation. **Nausheen Ahmed:** Writing – review & editing, Visualization, Validation, Supervision, Conceptualization. **Forat Lutfi:** Writing – review & editing, Visualization, Validation, Supervision, Investigation, Conceptualization. **Muhammad Mushtaq:** Writing – review & editing, Visualization, Validation, Supervision, Conceptualization. **Leyla Shune:** Writing – review & editing, Visualization, Validation, Supervision, Investigation. **Anurag Singh:** Writing – original draft, Visualization, Validation, Supervision, Resources. **Sunil Abhyankar:** Writing – review & editing, Visualization, Validation, Supervision, Investigation. **Joseph McGuirk:** Writing – review & editing, Visualization, Validation, Supervision, Conceptualization. **Haitham Abdelhakim:** Writing – review & editing, Visualization, Validation, Supervision, Resources, Project administration, Conceptualization.

## Declaration of competing interest

The authors declare that they have no known competing financial interests or personal relationships that could have appeared to influence the work reported in this paper.

## Appendix Supplementary materials

Image, application 1
