# Supplementary material for: Delayed fatal neurotoxicity in post CAR-T cell therapy for multiple myeloma, a case report
Source: Leuk Res Rep. 2026 Mar 3;25:100577. doi: 10.1016/j.lrr.2026.100577 (PMC12991952; doi:10.1016/j.lrr.2026.100577)
Supplement: Supplementary file 1 [file mmc1.docx]

**Supplementary Material**

**Supplemental Figure 1**

Supplementary Figure 1: Ferritin levels through case timeline in ng/ml.
